# Justification and Optimization Principles of ALARA in Pediatric CT at a Teaching Hospital in Ethiopia

**DOI:** 10.4314/ejhs.v30i5.16

**Published:** 2020-09

**Authors:** Daniel Z Solomon, Bezawit Ayalew, Seife T Dellie, Daniel Admasie

**Affiliations:** 1 Department of Radiology, College of Health Sciences, Addis Ababa University

**Keywords:** pediatric CT, radiation dose, optimization, Justification

## Abstract

**Background:**

Radiation from CT (computerized tomography), poses risk of radiation associated cancer. Studies indicate a cumulative dose of 50mGy triples the risk of leukemia and a dose of 60mGy triples the risk of brain tumors in children. This study aimed to assess the application of “Justification and Optimization Principles of ALARA (As Low As Reasonably Achievable)” in pediatric CT.

**Method:**

A prospective cross-sectional study was conducted from December 2017 to July 2018 G.C at Tikur Anbessa Specialized Hospital. CT request forms were reviewed. All pediatric patients listed for CT were included. The collected data were analyzed using SPSS version 25.

**Results:**

Four hundred and twenty nine CT requests were reviewed, 246 (57.3%) were males and 183(42.7%) were females; 52(12.1%) were less than 1 year old, 153(35.7%) between 1 to 5 years, 113(26.3%) between 5 to 10 years and 111(25.8%) 10 to 14 years old. On the other hand, 28(6.5%) scan requests were rejected based on the ALARA justification principle, and from these, in 11(39.2%) MRI was recommended instead of CT, in 6(21.42%) US was recommended. Review of previous CT helped to reject 4(14.28%). Review of prior chest radiographs helped in rejecting 2(7.14%) requests. For 5(17.8%) and 19(4.4%), requests were optimized by applying principles of optimization to reduce received dose from CT.

**Conclusion:**

Overall, 47(11%) patients were protected from unnecessary radiation exposure by applying the principles of ALARA. The use of other alternating imaging modalities is vital in pediatric patients who are more radiosensitive and have longer time to manifest radiation induced injury.

## Introduction

Exposure of tissues or organs to ionizing radiation can induce the death of cells on a scale that can be extensive enough to impair the function of the exposed tissue or organ. Effect of this type is called ‘deterministic effect’, which is clinically observable in an individual only if the radiation dose exceeds a certain threshold (1).

Exposure to radiation can also induce a non-lethal transformation of cells, which may still retain their capacity for cell division. The human body's immune system is very effective in detecting and destroying abnormal cells. However, there is a possibility that the non-lethal transformation of a cell could lead, after a latency period, to cancer in the individual exposed, if it is a somatic cell, or to hereditary effects, if it is a germ cell. Such effects are called ‘stochastic’ effects (1).

Radiation exposure, particularly with CT imaging, continues to be worrisome for children who are more vulnerable than adults from radiation-associated cancer development. In fact, a recent study indicated that a cumulative dose of 50 mGy triples the risk of leukemia, and a dose of 60 mGy triples the risk of brain tumors in children. Although CT represents only10 to 15% of all imaging, the radiation risk from CT is highest in diagnostic imaging contributing up to 67% of all radiations (2).

The pediatric population is a very inhomogeneous group broadly encompassing those aged 0–18 years. Growing tissues are more sensitive to the mutagenic effect of ionizing radiation. The younger the child, the larger the number of growing cells. Using the current system of risk assessment, the risk of developing a solid tumor after radiation exposure is about 3 times higher for a 1-year-old child and 1.8 times higher for a 10-year-old child compared to adults. Gender also influences risk-females are exposed to a further 50% increase in relative risk owing to the higher radio sensitivity of breast tissue and the associated incidence of breast cancer (3).

Although overall pediatric CT utilization accounts for 8–10% of all CT examinations in the USA, according to a study by Gonzalez et al, the trend or tendency of employing CT is getting higher in pediatric than in adult populations (4). The International Commission for Radiation Protection (ICRP) has recommended a system of dose limitation composed of the following requirements: justification of practices involving radiation exposures, optimization of the level of protection for such practices and individual dose limitation (5). As Low As Reasonably Achievable (ALARA) represents a practice mandate adhering to the principle of keeping radiation doses to patients and personnel to acceptable levels. This concept is strongly endorsed by the Society for Pediatric Radiology, particularly in modalities involving higher radiation doses (CT and fluoroscopy) (6,7).

A study conducted to compare the dose-length-product and effective radiation dose to patients from CT examinations revealed a considerable variation, justifying the need to optimize the effective dose to the patient (8). Another study conducted to explore the risks of low-level radiation and CT found a statistically significant, increased risk of fatal cancer from low-dose radiation in the range of 50 to 100 mSv. For example, a single CT of the abdomen could provide a dose of 11 mSv. If there are 3 phases in this examination, the actual dose will be 33 mSv. If a child receives 3 or more examinations, he will have received a dose equivalent to the lifetime dose of approximately 100 mSv, a range associated with induction of fatal cancer (9,10). The National Cancer Institute suggested several steps to reduce the radiation dose to children such as only necessary CT examinations be performed, and if CT is the appropriate modality, exposure parameters should be optimized (limiting the region of interest, mA settings and lower resolution) (11). Another study suggested the use of automatic exposure settings, guidelines to compare dose indicators with standards, employ DICOM (Digital Imaging and Communications in Medicine) file formatting, and personnel training (12). A Finish study to assess the prevalence of unjustified CT imaging in younger patients showed that about 30% of all examinations were unjustified (13).

The practice of the ALARA principle in the developed world is currently well established. However, there is a striking lack of published data regarding such experience in Africa, and particularly in Ethiopia, thereby necessitating the need for this study. The objective of this study is mainly to assess the clinical application of justification and optimization principles of ALARA in pediatric CT imaging. We believe that this study, in the absence or scarcity of information at local and regional (African) levels, will shade light as to whether justification and optimization of pediatric CT imaging requests would indeed help in reducing unnecessary radiation in children.

## Methods and Materials

The study was conducted at Tikur Anbessa Specialized Hospital (TASH), College of Health Science, Addis Ababa University, Addis Ababa, Ethiopia as an institution-based prospective cross-sectional study from December 2017 to July 2018. The source population was all pediatric patients who visited the hospital with the study population as those patients who were sent to the Radiology Department for CT imaging during the study period.

A non-random convenience sampling method was used, and all pediatric patients who came for CT imaging in the study period were included in the study. Patients who were ≤ 14 years old and scanned during regular hours were included, while those who came during duty hours were excluded for reasons of poor communication and incomplete clinical information.

Data were collected using a standardized CT request form with preset form fields which had to be completed manually by the physician requesting the CT imaging. Completeness and adequacy of the data in CT request forms were ensured in all cases. We assessed if the history provided was adequate for justifying the scan requested by utilizing standard imaging indications for the given clinical setting, and where information was felt to be inadequate, direct communication with the requesting physician was made, or information from the patients' files were sought including previous radiological investigation. The data collected were analyzed using SPSS version 25 statistical package. Ethical clearance and permission were obtained from the IRB. The pediatric CT protocol of the department was used -(single phase post contrast CT for all pediatric patients) except in cases with fracture, urinary stone, foreign body aspiration, and high-resolution temporal CT (where pre-contrast CT is done), and for liver masses where tri-phasic CT is needed and CT urography for genito-urinary pathology are performed. For the purpose of our procedure, the ALARA principles were used for reviewing CT requests as shown below.

**Justification of medical exposures**: All medical imaging exposures must show a sufficient net benefit when balanced against possible detriment that the examination might cause.

**Optimization**: It is based on the standard pediatric CT protocol. It is a process of evaluation of image quality against patient dose and opting for possible alternatives to maintain necessary image quality while minimizing patient-absorbed doses, or selection of better imaging protocols under the given circumstances, and implementation of the selected option and regular review of image quality and patient dose to evaluate if either requires further action.

## Results

Among the total of 429 CT requests received during the study period, 246(57.3%) were males and 183(42.7%) were females. Age distribution showed 52(12.1%) as less than 1 year, 153(35.7%) were in the 1–4-year group, 113(26.3%) were between 5–9 years and 111(2.9%) were in the 10–14-year group (Table 1). Eighty-one (18.9%) requests were received from the in-patient unit, 127(29.6%) were received from the outpatient section, 35(8.2%) were received from the Emergency Department, and in 186(43.4%), the unit was not mentioned. Out of the total requests received, 424(98.8%) had complete clinical data documented on the request form while 5(1.2%) had incomplete clinical data from which for 3, the requesting physicians were communicated and for 2, patients' charts were reviewed.

**Table 1:** Age distribution of pediatric patients sent for CT scan at Tikur Anbesa Hospital, Addis Ababa, Ethiopia, December 2017–July 2018

Age (years)	Frequency	Percentage
**<1**	52	12.1%
**[1–4)**	153	35.7%
**[5–9)**	113	26.3%
**[10–14]**	111	25.9%
**Total**	429	100%

**Figure 1:**
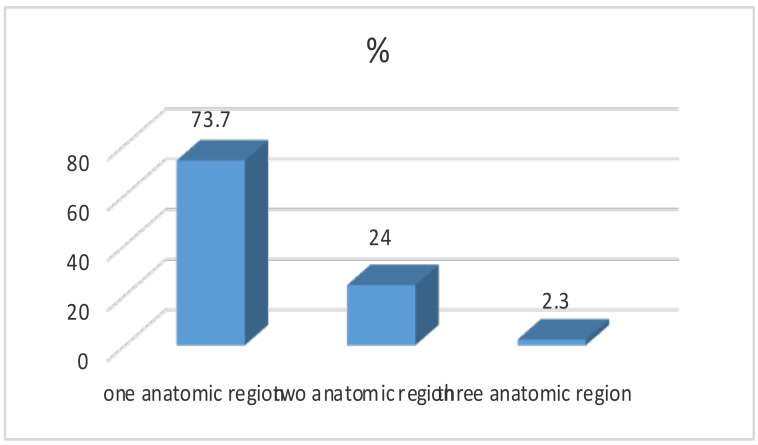
Distribution by number of anatomic regions of pediatric patients referred for CT at Tikur Anbessa Hospital, Addis Ababa, Ethiopia, December 2017–July 2018

Of the total requests, 316(73.7%) were for one anatomic region, 103(24%) for two anatomic regions and 10(2.3%) for three anatomic regions (Figure 1). Most of the CTs performed were for the abdomen, 154(35.9%), while scans for the spine and musculoskeletal regions were scanty with 3(0.7%) each. The remaining requests included head CT, 111(25.9%), neck CT, 52(12.1%), PNS, 12(2.8%), temporal, 6(1.4%), chest, 126(29.4%) and pelvic, 38(8.9%).

**Figure 2:**
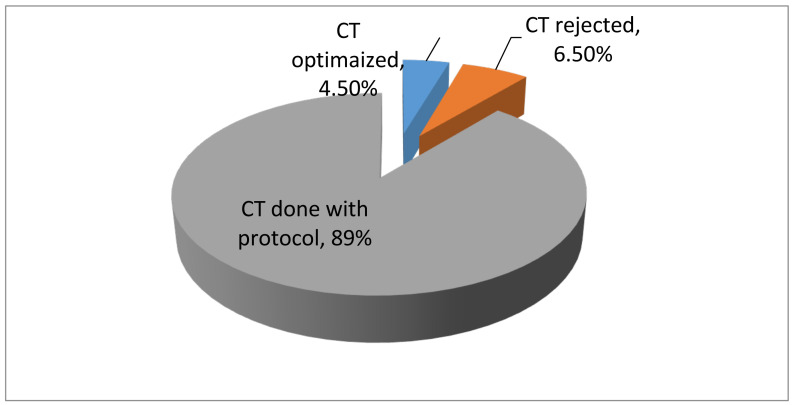
Distribution of CTs modified, rejected and done with protocol for pediatric patients seen in Tikur Anbessa Hospital, Addis Ababa, Ethiopia, December 2017–July 2018

From the 429 requests, 28(6.5%) were rejected based on ALARA justification principle. Multiple reasons for the rejected requests were seen. In 11(39.2%) of the requests, MRI was recommended instead of CT and in 6(21.42%), US was advised instead of CT. Additionally, review of previous CT scan helped to reject 4(14.28%) requests. Review of prior chest radiography helped in rejecting 2(7.14%) requests. For 5(17.8%), other imaging modalities like endoscopy, echocardiography, IVU and barium fluoroscopy studies were recommended (Table 2). Nineteen (4.5%) CT requests were optimized by ALARA optimization principles (Figure 2).

**Table 2:** Reasons for optimization and rejection of CT scans requested for pediatric patients in Tikur Anbesa Hospital, Addis Ababa, Ethiopia, December 2017–July 2018

Reason for optimizing or justifying/rejecting CT	Frequency	Percentage
US could provide needed information	6/ justified rejection	1.4%
MRI could be used instead of CT	11/ justified rejection	2.6%
Review of previous imaging studies was sufficient	4/ justified rejection	0.9%
CXR reviewed was able to provide necessary information	2/ justified rejection	0.5%
One area scan was enough with review of other imaging	14/optimized	3.3%
Collimated to specific area of interest	5/optimized	1.2%
Other investigations like IVU, Barium, Echo and endoscopy were preferred	5/justified rejection	1.2%

Eight (15.3%) out of 52 children were in the less-than 1-year group. 16(10.4%) out of 153 children were between 1 and 4 years, while 14(12.3%) out of 113 children were between ages 5 and 9 years old. 9(8.1%) out of 111 children between ages 10 and 14 were protected from unwanted radiation exposure through employment of justification and optimization principles of ALARA (Table 3).

**Table 3:** Age-wise distribution of requested and modified or rejected CT scans of pediatric patients seen in Tikur Anbessa Hospital, Addis Ababa, Ethiopia, December 2017–July 2018

Age (years)	Frequency	Number of CT rejected or optimized	Percentage
**<1**	52	8	15.3%
**[1–5)**	153	16	10.4%
**[5–10)**	113	14	12.3%
**[10–14]**	111	9	8.1%

## Discussion

In our department, CT protocol for pediatric patients is only post contrast single-phase CT scan with exceptions such as trauma to identify fractures, hematomas in brain imaging or ureteric stones where pre-contrast CT is required including high-resolution temporal CT. The use of pre- contrast CT scan ever results in clinically relevant extra information and should usually be abandoned and in the same manner, multiphase CT scan examination should be avoided (4).

In some cases, where mass is identified on US, tri-phasic abdominal CT for further characterization is requested. In these cases, physicians usually write triphasic abdominal CT on the request paper since they have the recommendation from the abdominal US. In most other cases, they write only the anatomic regions to be scanned and the request papers are protocolled in the pediatric radiology and neuroradiology units.

Since only single phase (which is only post contrast) CT is done for almost all patients with some exceptions as described above, it is not considered as optimization in this study.

In our study, the percentage (11%) of CT requests either modified or rejected using optimization or justification principle of ALARA were more or less comparable with a similar Indian study with 8.06% (2). The Indian study considers changing the requests from two phase to one phase as optimization, while in our study, this was not taken as optimization.

Our rejection result (6%) was also in agreement with the Indian study which showed 4.6%. From our rejected requests, MRI and US were recommended instead of CT for about 2/3 of requests, while in the Indian study, US and MRI were suggested for 15(13.5%) (2).

In our study, the main reason for either optimization or rejection was found to be one -area-scan with review of previous imaging. An Indian study showed the main reason for optimization or rejection to be single-phase CT as sufficient to answer the clinical question (2).

A Finish study, on unjustified CT examinations in young patients showed unjustified scans in 77% for the lumbar spine, 37% for the abdomen, 36% for the head and 3% for the cervical spine (3). More than two-third of requests had one anatomic region requested, a quarter had two anatomic regions requested and quite a few (2.3%) had three anatomic regions requested. In contrast, the Indian study (2) revealed 1216(93.4%) CT requests for one anatomic region, 82(6.3%) for two anatomic regions and 4(0.3%) for three anatomic regions requested with the maximum number of requests received being for CT of head (780/56.03%) while in our study, the maximum number of requests were for CT of abdomen in about a third of requests.

The results of this study are in agreement with other similar studies cited above in showing that a significant number of pediatric patients can be protected from unnecessary or additional radiation exposure from CT imaging when justification and optimization principles of ALARA are applied before obtaining CT imaging. In conclusion, our study has shown that a considerable portion of our pediatric patients were protected from unnecessary radiation exposure by applying the principles of ALARA with obvious advantage in decreasing radiation exposure to children. The use of other alternating imaging modalities like US or MRI with no radiation exposure has a major role in replacing CT in the pediatric population who are more radiosensitive and have longer life time to manifest radiation induced injury.

A mandatory protocol for a detailed clinical data, specific indication for imaging, and request for previous imaging reports (if any) should be placed on the request form. Imaging departments should take the initiative to create awareness about the risk of radiation in pediatric patients and the value of alternative imaging modalities in their clinical settings through regular joint clinical inter-disciplinary panels. Health provision authorities should play a leading role in facilitating and enhancing proper practice and consider planning for other alternative imaging modalities with no radiation risk in all higher-level medical facilities.
